# Functional dissection of *Corynebacterium glutamicum* Wag31 domains for septal recruitment and polar distribution during the cell cycle

**DOI:** 10.1128/mbio.01153-26

**Published:** 2026-06-22

**Authors:** Julienne Petit, Daniela Megrian, Mariano Martinez, Adrià Sogues, Gabriel Brunk, Célia de Sousa-d'Auria, Mathilde Ben Assaya, Catherine Thouvenot, Emilie Lesur, Yann Bourdreux, Nicolas Bayan, Pedro M. Alzari, Anne Marie Wehenkel

**Affiliations:** 1 Bacterial Cell Cycle Mechanisms Unit, Institut Pasteur, Université Paris Cité, CNRS UMR 3528 27058, Paris, France; 2 Structural Microbiology Unit, Institut Pasteur, Université Paris Cité, CNRS UMR 3528, Paris, France; 3 Université Paris-Saclay, CEA, CNRS, Institute for Integrative Biology of the Cell (I2BC), Gif-sur-Yvette, France; 4 Ultrastructural BioImaging Unit, Institut Pasteur, Université Paris Cité, Paris, France; 5 Université Paris-Saclay, CNRS UMR 8182, Institut de Chimie Moléculaire et des Matériaux d'Orsay, ICMMO, Orsay, France; Indiana University Bloomington, Bloomington, Indiana, USA

**Keywords:** Wag31, morphogenesis, elongasome, Actinobacteria, polar growth, cell cycle, coiled-coil domain, DivIVA

## Abstract

Bacterial cell morphogenesis is controlled by the synthesis and organization of peptidoglycan and driven by multi-protein complexes, such as the divisome and elongasome. Here, we investigate the role of the *Corynebacterium glutamicum* DivIVA homolog*,* Wag31, the elongasome scaffold that is required for polar growth in *Corynebacteriales*. Conditional depletion of Wag31 results in coccoid-shaped cells, able to divide over several generations, showing that Wag31 is essential for rod shape maintenance, but not for viability in the conditions tested. Wag31/DivIVA homologs are formed by two distinct coiled-coil domains, a highly conserved N-terminal domain (NtD, also referred to as the DivIVA domain), and a more variable C-terminal domain (CtD). Our structural phylogenetic analyses revealed that in *Actinobacteria*, unlike *Firmicutes*, an intrinsically disordered region spatially separates the NtD from the CtD. We show that the NtD is necessary and sufficient for septum localization, while the CtD mediates self-interaction and polar accumulation. Our findings suggest that Wag31 is recruited specifically to the septum through protein–protein interactions involving the NtD, priming the future pole, and allowing for a timely divisome-elongasome transition at cytokinesis. Once the pole is formed, the self-aggregative properties of the CtD dominate and form a stable structure that likely organizes the pole for cell wall biosynthesis.

**IMPORTANCE:**

Wag31, the elongasome scaffold in *Corynebacteriales*, is a cytoskeletal protein that remains poorly understood. In *Firmicutes,* the Wag31 homolog, DivIVA, has been largely studied, but its function differs from that of *Actinobacteria*. Using AlphaFold predictions and evolutionary analysis, we found a clear sequence and structure cutoff between *Firmicutes* and *Actinobacteria*, correlating with their different functions. An intrinsically disordered insertion in Wag31 appeared early in the actinobacterial tree, separating the membrane/septum binding function of the N-terminal DivIVA domain from the C-terminal coiled-coil domain’s self-aggregation function. We analyzed each domain’s role and assigned a dominant role in cellular distribution. The N-ter is necessary for septal recruitment, independent of membrane binding capacities. Timely recruitment is essential for correct cellular distribution, as interference with this domain leads to asymmetric Wag31 localization and septum misplacement. We propose that Wag31’s early role at the septum is to prime the future pole, where it organizes polar growth upon cytokinesis.

## INTRODUCTION

Bacterial cell shapes are driven by the synthesis of peptidoglycan (PG), a three-dimensional polymer formed by long glycan strands cross-linked via short peptide chains (1). Cell wall biogenesis needs to be finely regulated during the cell cycle to allow for the generation of two identical and viable daughter cells, and thus, bacterial morphogenesis relies on the tight coordination between cell division and elongation. These two processes are precisely orchestrated by large multi-protein complexes, the divisome and the elongasome, respectively, that drive the assembly of the bacterial cell wall and guide PG insertion (2). The organization and assembly of these machineries generally rely on essential cytoskeletal proteins. Divisome assembly requires FtsZ, the nearly universally conserved tubulin homolog. However, the elongasome relies on different scaffolds in different bacteria (3). This diversity reflects the specific modes of bacterial cell elongation, which can proceed from the lateral side (dispersed growth), the cell center (septo-peripheral growth), or the cell pole (polar growth) (4). The dispersed lateral growth, driven by the actin-like MreB protein to organize the Rod complex, has been well characterized in model bacteria like *Escherichia coli* (5) or *Bacillus subtilis* (6, 7). MreB has been extensively studied, and detailed molecular mechanisms underlying its function have been described (8). By contrast, less is known about the molecular determinants required for cell elongation in bacteria without lateral growth, which lack MreB and most of the Rod complex orthologs, except for the enzymatic couple between RodA, the glycosyltransferase (GT) (a Shape, Elongation, Division, and Sporulation [SEDS]-like protein), and its cognate transpeptidase, a class B penicillin-binding protein (b-PBP) (9). This is notably the case for polar-growing rod-shaped *Corynebacteriales*, an important suborder of *Actinobacteria* that contains major human pathogens, such as *Mycobacterium tuberculosis* or *Corynebacterium diphtheriae*. In these bacteria, the coiled-coil scaffolding protein Wag31 (DivIVA) is crucial for polar growth (10, 11).

Wag31/DivIVA is broadly conserved in Gram-positive bacteria (12), and in both *Firmicutes* and *Actinobacteria*, it localizes to the poles and the division site, where it is thought to sense negative curvature (13, 14). In most *Firmicutes*, DivIVA is a non-essential protein responsible for polar localization of the MinCD complex, thereby preventing the formation of the division site at the poles (15, 16). In polar-growing *Actinobacteria*, Wag31/DivIVA is an essential protein required for elongation and is thought to be the scaffold of the PG synthesis machinery (11, 17, 18). For instance, in *Streptomyces coelicolor,* DivIVA is part of the polarisome that localizes to the tips of the hyphae and future sites of branching, where it promotes PG synthesis (19). Branching appears over the lateral walls, where new foci of DivIVA accumulate. Its overexpression causes hyperbranching, and the tips become swollen and rounded (11). In *Mycobacteria*, Wag31 (also known as Antigen 84) localizes asymmetrically to the cell poles, as well as to the septum, and this localization correlates with the incorporation of nascent PG (18). Indeed, PG labeling in a conditional Wag31 depletion strain showed that PG can still assemble in the absence of Wag31 but is delocalized and dispersed around the cells (20). Wag31 is also thought to direct the assembly of the outer layers of the complex mycobacterial cell envelope (composed of three distinct macromolecules—peptidoglycan, arabinogalactan, and mycolic acids) at the pole by organizing the inner membrane domain through direct or indirect interaction with enzymes involved in the early steps of cell wall precursor synthesis (21–24). However, it is unclear if Wag31 plays a direct role in membrane organization or is just a consequence of localized membrane synthesis (25). In *Corynebacterium glutamicum*, Wag31 overexpression leads to polar bulging, and its depletion to cellular rounding (10). Wag31 colocalized with the SEDS GT RodA of the elongasome in cellular interaction assays (26, 27). Apart from its role in polar elongation, Wag31 has also been shown to associate with the chromosome segregation machinery through direct or indirect interaction with ParB (28).

Wag31/DivIVA is a coiled-coil protein defined by a highly conserved 60 amino acid N-terminal domain (NtD), also referred to as the DivIVA-domain (29), followed by a less conserved, longer C-terminal domain (CtD), mostly formed of coiled-coils and intrinsically disordered regions of variable length. The NtD folds into a dimeric parallel coiled-coil capped with two intertwined loops at one end that expose hydrophobic and positively charged residues. This domain was reported to interact with membranes in Wag31 orthologs from *B. subtilis* (14), *Clostridium difficile* (30), and *M. tuberculosis* (24). The CtD domain was shown to form a long curved coiled-coil tetramer in *B. subtilis* (14), reminiscent of BAR-like domains, which are long (20 nm) arc-shaped coiled-coil structures capable of sensing and remodeling membrane curvature in eukaryotes (31). However, almost 20 years after the first oligomers were observed in *B. subtilis* (32), the precise structural arrangement of Wag31/DivIVA oligomers and their role during the cell cycle remain elusive.

In this work, we set out to understand the molecular function of the individual Wag31 N- and C-terminal domains in *Corynebacterium glutamicum*. We generated a conditional depletion strain for cellular studies and showed that this depletion resulted in round cells that divide and have a fully formed cell envelope. Phylogenetic studies show that Wag31 has acquired an intrinsically disordered domain that structurally separates the NtD from the CtD. Using cellular localization and complementation studies, we identified distinct functional roles for each domain, as the NtD is necessary and sufficient for septum localization, likely mediated by protein–protein interactions, while the CtD is mainly responsible for self-interaction and polar protein accumulation. Interfering with the NtD systematically leads to asymmetric distribution of Wag31 to one pole, suggesting that correct priming at the septum prior to cytokinesis is essential for cellular repartitioning of the elongasome scaffold. Together, these results uncover new roles and *Corynebacteriales*-specific mechanisms for this crucial protein, paving the way for detailed mechanistic studies.

## RESULTS

### A conditional Wag31 depletion strain is coccoid-shaped and viable over several generations

**Fig 1:**
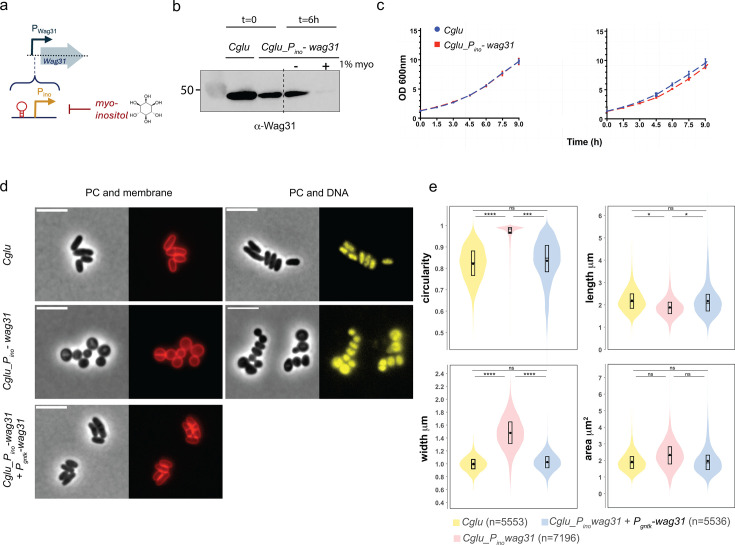
Phenotypic characterization of *Cglu_P_ino_-wag31*. (**a**) Schematic description of depletion strategy. (**b**) Western blot of whole cell extracts (60 μg) from *Cglu* and *Cglu_P_ino_-wag31* after overnight growth in minimal medium (*t* = 0) and grown for 6 h in the absence or presence of 1% *myo*-inositol (*t* = 6 h). Wag31 levels were revealed using anti-Wag31 antibodies. (**c**) Growth curves of *Cglu* (blue) and *Cglu_P_ino_-wag31* (red) grown in minimal medium with 1% *myo*-inositol monitored during the first 9 h (left panel). After 24 h, the cultures were diluted a second time in fresh medium with 1% *myo*-inositol and grown for another 9 h (right panel). (**d**) Representative images in phase contrast (PC) and Nile Red (membrane) on the left and PC and Hoechst (DNA) on the right for *Cglu*, *Cglu_P_ino_-wag31,* and *Cglu_P_ino_-wag31* complemented with *P_gntK_-wag31* (+ gluconate) strains grown depleted of Wag31 for 6 h in minimal medium. (**e**) Violin plots showing the distribution of cell circularity, cell length, cell width, and cell area for *Cglu*, *Cglu_P_ino_-wag31,* and *Cglu_P_ino_-wag31 + P_gntK_-wag31* (+ gluconate) depleted for 6 h. Cohen’s *d* for circularity: (^ns^*d* = 0.22), (^***^d = 1.79), (^****^d = 2.46); for length: (^ns^*d* = 0.12), (left, ^*^d = 0.76), (right, ^*^d = 0.54); for width: (^ns^*d* = 0), (left, ^****^d = 2.26), (right, ^****^d = 2.18); for area: (top, ^ns^*d* = 0.07), (left, ^ns^*d* = 0.44), (right, ^ns^*d* = 0.46).

In *Corynebacteriales*, *wag31* is defined as an essential gene (17) found at the end of the division and cell wall (dcw) cluster. To understand the role of Wag31 in the cell, we designed a conditional depletion strain (*Cglu_P_ino_-wag31*) in *Corynebacterium glutamicum* (*Cglu*, ATCC13032) by placing a transcriptional terminator just before the *wag31* open reading frame, and by putting it under the control of the *myo*-inositol-repressible promoter (*P_ino_*) of the inositol phosphate synthase *Ino1* gene (33) (Fig. 1a). Wag31 protein levels were assessed after 6 h by western blot, revealing an almost complete depletion of the protein (Fig. 1b). Surprisingly, depleted cells were viable after 24 h of growth and kept growing in a second exponential phase in the presence of *myo*-inositol following an overnight depletion and re-dilution (Fig. 1c). To investigate the impact of Wag31 depletion on cell morphology, we imaged the *Cglu_P_ino_-wag31* strain in the absence and presence of *myo*-inositol. As expected from previous work (10, 34), depleted cells went from a rod-shaped to a coccoid morphology (Fig. 1d). Despite this dramatic change in shape, the bacteria still formed septa and segregated DNA between daughter cells (Fig. 1d), suggesting a fully functional divisome machinery. The depleted cells display a circularity close to 1 (0.98) (Fig. 1e), their length decreases (from 2.24 ± 0.53 µm to 1.88 ± 0.43 µm and 1.62 ± 0.32 µm after 6- and 24-h depletion, respectively), and width increases from 1.03 ± 0.10 µm to 1.48 ± 0.25 µm, which results in a mostly unchanged cell area (Fig. 1e). To verify that the observed phenotype was solely due to the depletion of Wag31, we expressed Wag31 ectopically from a plasmid under the control of *P_gntK_*, a tight promoter that is repressed by sucrose (4%) and induced by gluconate (1%) (35). Unless otherwise stated, in the following experiments, we used minimal medium (CGXII, see Materials and Methods) supplemented with 4% sucrose, where Wag31 expression is minimal (Fig. S1), or 1% gluconate/4% sucrose, where Wag31 is overexpressed, and expression levels are close to native (Fig. S1) . The Wag31 depletion phenotype was rescued when the strain was grown for 6 h in the presence of gluconate (Fig. 1d and e). Circularity was 0.84 ± 0.10, a value similar to the WT (0.82 ± 0.08) (Fig. 1e), and length and width also went back to WT-like values (Fig. 1e).

### A full cell envelope is assembled in the absence of Wag31

**Fig 2:**
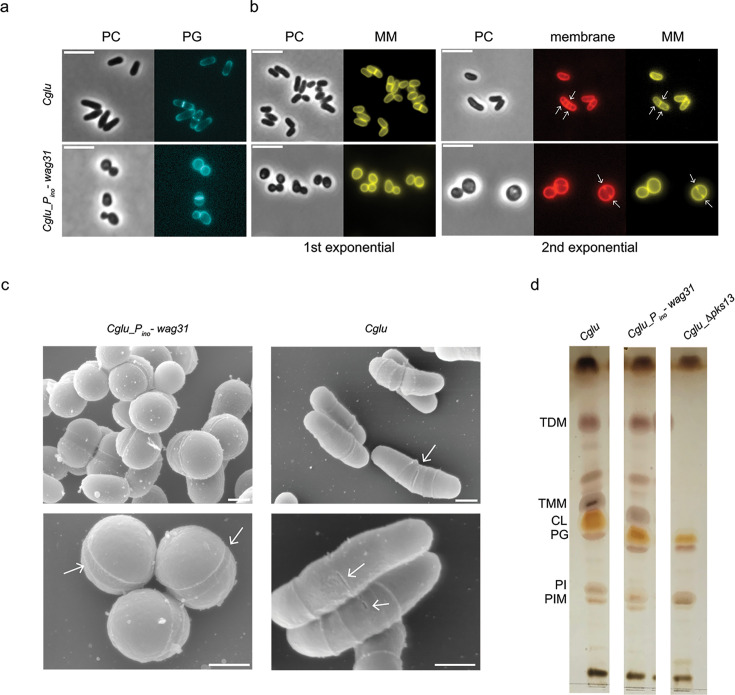
Characterization of the cell envelope of the depleted *Cglu_P_ino_-wag31* strain. (**a**) Representative images in phase contrast (PC) and HADA (PG, blue) for *Cglu* (top left) and *Cglu_P_ino_-wag31* (bottom left) depleted for 6 h. (**b**) Representative images in phase contrast, Nile Red (membrane, red), and 2-FIC5Tre (mycomembrane, MM, yellow) staining, for *Cglu*, and depleted *Cglu_P_ino_-wag31* in a first and second (rediluted from the first) culture. White arrows indicate forming septa (red channel) and TMM incorporation (yellow channel). Note that 2-FIC5Tre staining was observed in the depleted cells at the nascent septum (white arrows, bottom panel), which differs from the previously reported WT situation where the glycolipids infiltrate the septum just before the mechanical V-snap (37). Scale bars: 5 μm. (**c**) Scanning electron micrographs showing depleted *Cglu_P_ino_-wag31* (top) *and Cglu* (bottom) at magnifications of 27,000× and 50,000×. Scale bars: 500 nm. Arrows indicate cleavage furrows for *Cglu_P_ino_-wag31* and pre-rupture holes for *Cglu*. (**d**) TLC analysis of crude lipid extracts isolated from cells grown in 1% *myo*-inositol in minimal medium to the stationary phase. Comparable amounts were loaded on TLC plates for Cglu, *Cglu_P_ino_-wag31* depleted cells, or Δ*pks13.* PG, phosphatidylglycerol; CL, cardiolipin; PI, phosphatidylinositol; PIM, phosphatidylinositol mannosides; TMM, trehalose monomycolates; TDM, trehalose dimycolates.

To understand a possible direct role for Wag31 in regulating elongasome function and cell envelope assembly, we first assessed cell wall composition in the depleted *Cglu_P_ino_-wag31* and the *Cglu* strains. Wag31-depleted cells incorporate PG at the septum in line with a functional divisome (Fig. 2a). To evaluate the integrity of the mycomembrane (MM), we stained the bacterial cells with 2-FIC5Tre (36), a trehalose-based probe that is converted by mycoloyltransferases into a fluorescent version of trehalose monomycolate (TMM), a key glycolipid of the outer membrane. The depleted *Cglu_P_ino_-wag31* cell surface was fully stained by the probe (Fig. 2b). To rule out the possible incorporation of mycolic acids before complete Wag31 depletion, we grew *Cglu_P_ino_-wag31* cells overnight in *myo*-inositol, then initiated a second exponential phase with pre-depleted cells in fresh medium to eliminate residual mycolic acids. Even under these conditions, *Cglu_P_ino_-wag31* retained an MM similar to *Cglu* (Fig. 2b). Consistent with prior studies, glycolipids are incorporated after septum formation (Fig. 2b, white arrows, top panel), and the uniform cell surface staining is probably due to the high fluidity of the MM of *Cglu* (37). The Wag31-depleted strains exhibited a surface similar to WT cells in scanning electron micrographs (SEM, Fig. 2c). Interestingly, the rounded cells displayed deep cleavage furrows, likely marking active division sites, which differs from WT cells where characteristic holes (Fig. 2c, arrows) allow for glycolipid infiltration prior to V-snapping and daughter cell separation (37). These observations suggest that coccoid-shaped cells devoid of Wag31 divide by invagination rather than mechanical V-snapping post-cytokinesis.

The lipid composition of the cell envelope of *Cglu* and depleted *Cglu_P_ino_-wag31* strains was comparable (Fig. 2d), both showing a similar signal for trehalose mono- and di-mycolates (TMM and TDM), the most abundant glycolipids of the outer mycomembrane generally referred to as “free mycolates” because they are not covalently linked to the AG-PG layer (38). In contrast, the mutant *Cglu* strain Δ*pks13* (39), which has no mycomembrane because it is unable to synthesize mycolic acids, has neither TDM nor TMM (Fig. 2d). Taken together, these observations suggest that enzymes producing and incorporating the full cell envelope are present and functional when Wag31 is depleted, although more subtle differences in lipid homeostasis cannot be excluded, as recently shown for *Mycobacterium smegmatis* lipid composition upon Wag31 depletion (24).

### A structural phylogenetic analysis of Wag31/DivIVA paralogs reveals major differences between *Actinobacteria* and *Firmicutes*

**Fig 3:**
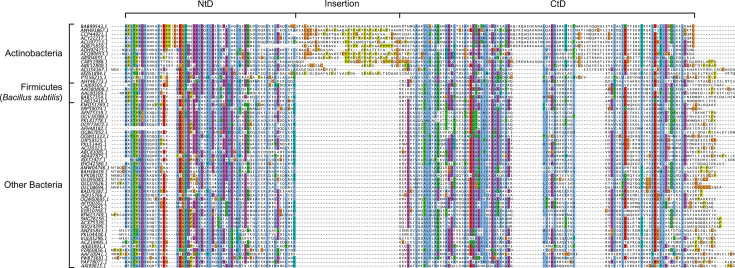
DivIVA/Wag31 family domain definition from sequence alignment. Trimmed multiple sequence alignment of DivIVA/Wag31 sequences identified in a database of representative species from 14 bacterial phyla. The boundaries of the protein domains (NtD and CtD) are indicated at the top. Sequences corresponding to *Actinobacteria, Firmicutes,* and other *Bacteria* are indicated on the left.

**Fig 4:**
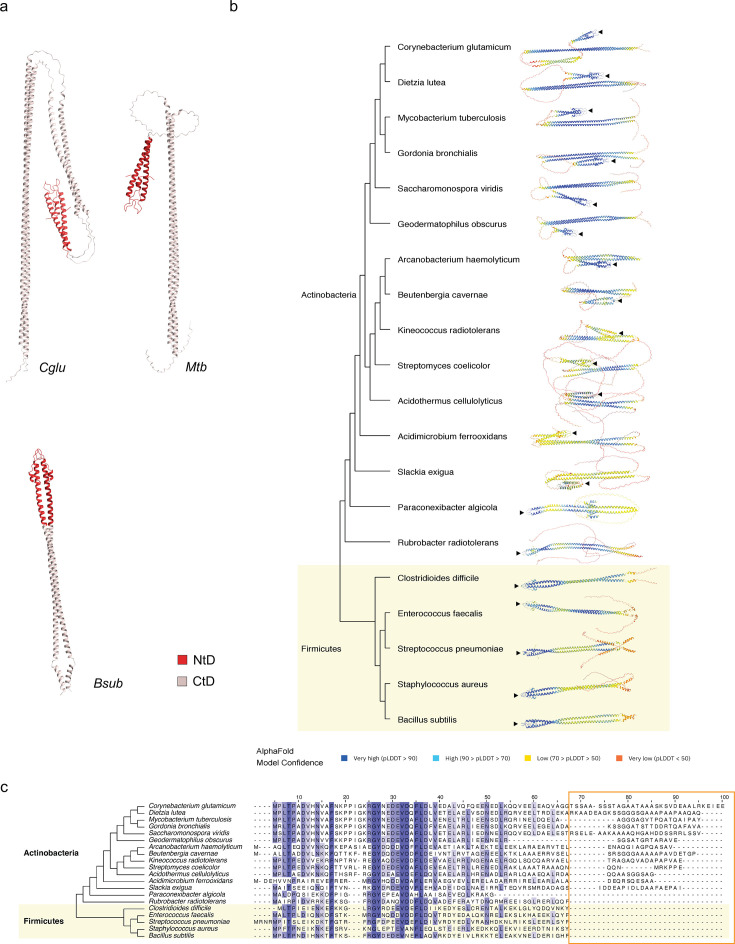
Structural and phylogenetic analysis of DivIVA/Wag31 domains. (**a**) AlphaFold prediction for dimeric full-length Wag31 homologs from *Cglu, Mtb,* and *Bsub*. (**b**) AlphaFold prediction for dimeric full-length Wag31 of representative species of Actinobacteria and Firmicutes, colored according to the AlphaFold model confidence and associated schematic phylogeny. The black arrow indicates the proximal tip of the conserved NtD that was shown to bind lipids in *B. subtilis*. (**c**) Schematic phylogenetic tree and multiple sequence alignment showing the sequences of the same representative species as in panel **b** and highlighting the insertion after the DivIVA-domain present in *Actinobacteria* (orange frame).

Wag31/DivIVA homologs display a highly conserved NtD or DivIVA domain connected to a CtD that is more variable in amino acid sequence and residue length (14, 34) (Fig. 3). For instance, in *Actinobacteria*, an insert can be observed at the boundary between NtD and CtD (residues 60–175, *Cglu* numbering) and others within the CtD domain, making these paralogs much longer than in other phyla (Fig. 3). Structural studies of full-length Wag31 are challenging as the protein tends to aggregate and form gel-like material during purification. This gel could be partially solubilized at high pH and high salt concentrations, but unsurprisingly, the protein did not yield protein crystals and did not result in interpretable images in cryo-EM. To obtain structural information on the full-length protein, we used AlphaFold (AF) to compute structural models of dimeric full-length Wag31 from *C. glutamicum* and *M. tuberculosis*, and DivIVA from *B. subtilis* (Fig. 4a). In the Wag31 models, the NtD is predicted to be spatially separated from the CtD by a disordered region that corresponds to the *Actinobacteria*-specific insert (Fig. 3). In contrast, the structure of *B. subtilis* DivIVA forms a continuous parallel coiled-coil dimer extending from the NtD into the CtD (Fig. 4a). To understand if this is a conserved feature of *Firmicutes*, we calculated structural models of representative DivIVA dimers for each order on the schematic phylogenetic tree shown in Fig. 4b. The evolutionary history, as well as the sequence alignment and structure predictions, strongly suggest that all *Firmicutes* DivIVA fold into a single continuous coiled-coil, whereas actinobacterial proteins (except for the most ancestral one, that may represent a transition) present a structurally disordered insert separating the NtD from the CtD (Fig. 3 and 4c). This acquired flexibility could allow for multiple conformational states and might explain in part the dynamic and functional differences between DivIVA in *Firmicutes* and *Actinobacteria* (40).

### Distinct functional properties of the Wag31 N- and C-terminal domains

**Fig 5:**
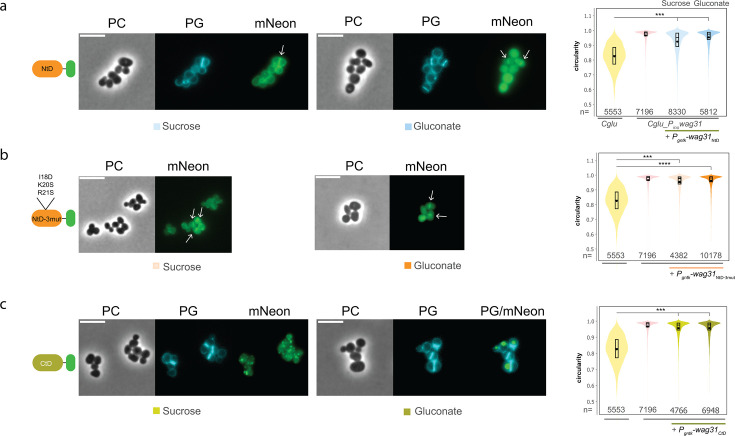
Domain characterization of Wag31_NtD_ and Wag31_CtD_. (**a**) Representative images in phase contrast (PC), HADA (PG), and green fluorescent signal (mNeon) in depleted *Cglu_P_ino_-wag31 + P_gntK_-Wag31*_Nt*D*_*-mNeon*. Low and high expression levels of Wag31_NtD_-mNeon in sucrose and gluconate, respectively. White arrows indicate the septal localization of Wag31_NtD_-mNeon. Violin plots showing the distribution of cell circularity in *Cglu* (WT), depleted *Cglu_P_ino_-wag31* (Pino 6h), and depleted *Cglu_P_ino_-wag31* expressing Wag31_NtD_-mNeon in sucrose without or with 1% gluconate (labeled sucrose or gluconate, respectively). Cohen’s *d*: (***, 1.25, 1.96). (**b**) Representative images in phase contrast (PC) and green, fluorescent signal (mNeon) in depleted *Cglu_P_ino_-wag31 + P_gntK_-Wag31_3mut_-mNeon*. Low and high expression levels of Wag31_NtD-3mut_-mNeon in sucrose and gluconate, respectively. White arrows indicate the septal localization of Wag31_NtD-3mut_-mNeon. Violin plots showing the distribution of cell circularity in *Cglu*, depleted *Cglu_P_ino_-wag31,* and depleted *Cglu_P_ino_-wag31* expressing Wag31_NtD-3mut_-mNeon in sucrose without or with 1% gluconate. Cohen’s *d*: (***, 1.81) (****, 2.25). (**c**) Representative images in phase contrast (PC), HADA (PG), and green, fluorescent signal (mNeon) in depleted *Cglu_P_ino_-wag31 + P_gntK_-Wag31*_CtD_*-mNeon*. Low and high expression levels of Wag31_CtD_-mNeon in sucrose and gluconate, respectively. The last panel corresponds to a PG/green signal overlay to better visualize the non-specific localization of Wag31_CtD_-mNeon foci. Violin plots showing the distribution of cell circularity in *Cglu*, depleted *Cglu_P_ino_-wag31,* and depleted *Cglu_P_ino_-wag31* expressing Wag31_CtD_-mNeon in sucrose without or with 1% gluconate. Cohen’s *d*: (****, 2.06, 2.15). All scale bars: 5 μm.

To understand the specific roles of the separate domains, we expressed fluorescently labeled truncation mutants containing only the NtD (Wag31_NtD_-mNeon) or the CtD (Wag31_CtD_-mNeon) in the *Cglu_P_ino_-wag31* strain. The *wag31* depletion phenotype was not rescued when the strain was complemented with Wag31_NtD_-mNeon (Fig. 5a). Cells were round and displayed similar circularity (0.95) to the depleted *Cglu_P_ino_-wag31* strain. Importantly, however, the NtD could still localize to the septum, where PG incorporation occurs (Fig. 5a), pointing to this domain as a driving force for septal localization of the full-length protein. As mentioned before, exposed hydrophobic and charged residues at the proximal tip of the *B. subtilis* DivIVA NtD were found to be essential for lipid binding (14). In our hands, however, we were unable to detect lipid binding of the *Cglu* NtD using a liposome sedimentation assay (Fig. S2), suggesting that NtD septal recruitment is mediated by protein–protein interactions. Moreover, a triple mutant (Ile18-Asp, Lys20-Ser, Arg21-Ser) of the equivalent hydrophobic and charged residues at the NtD tip in the Wag31_NtD_ construct (Wag31_NtD-3mut_) also retained the septal localization (Fig. 5b), suggesting that lateral NtD residues are primarily responsible for septal recruitment. We have previously shown that the membrane-associated Glp-GlpR complex mediates Wag31 recruitment to the mid-cell divisome *in vivo* (41) and that Wag31_NtD_ directly binds to GlpR *in vitro* with an apparent dissociation constant in the micromolar range (41), suggesting that this interaction could be a major driving force behind divisome recruitment and septal localization of Wag31.

The ectopic expression of Wag31_CtD_-mNeon in the depleted *Cglu_P_ino_-wag31* strain also failed to complement, but led, in a concentration-dependent manner, to a single aggregative spot randomly localized in the cell (Fig. 5c). The Wag31_CtD_-mNeon construct neither localized to the septum nor did it show any membrane localization but aggregated as a single spot in the cell, indicative of self-interaction. Although the structural basis by which Wag31 CtD homodimers can associate into higher-order oligomers in *Corynebacteriales* remains to be determined, high-confidence AlphaFold predictions suggest that two parallel coiled-coil dimers could form a tetramer in which the dimers are joined in the middle by a short anti-parallel four-helix bundle (Fig. S3), resembling similar arrangements previously seen in a low-resolution structure of *B. subtilis* DivIVA (14). Nevertheless, such interactions cannot account for the higher-order protein oligomers that likely occur at the poles and were indeed described to form in mycobacterial cell extracts (42). Additional oligomeric interactions may be driven by other regions of the coiled coils or the intrinsically disordered regions of the protein. In conclusion, our results suggest that the NtD of Wag31 is mainly responsible for specific divisome interactions and septum localization, whereas the CtD is primarily responsible for self-interaction and network formation at the poles of the WT cells. It should be noted that, although not assessed in our experiments, the possibility of specific interactions of Wag31 with other polar proteins might also play a role in polar assembly and function.

### An altered NtD domain leads to Wag31 polar asymmetry

**Fig 6:**
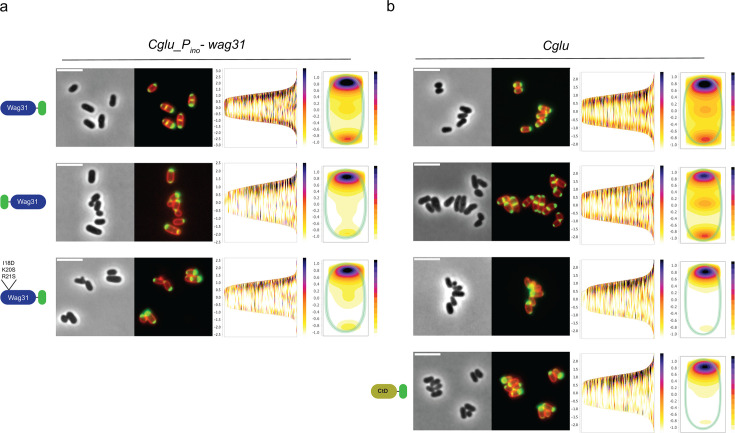
Asymmetric Wag31 localization when the NtD is compromised. (**a**) Representative images in phase contrast and superposed Nile Red and mNeon fluorescent signal in the depleted *Cglu_P_ino_-wag31* strain overexpressing the different constructs are represented on the left. Cell profile alignments (cells sorted by length) and heatmaps representing the localization pattern of each construct. (**b**) Representative images in phase contrast and superposed Nile Red and mNeon fluorescent signal in the *Cglu* strain overexpressing the different constructs are represented on the left. Cell profile alignments (cells sorted by length) and heatmaps representing the localization pattern of each construct. Cells in panel **a** were grown in minimal medium with 1% gluconate and 1% *myo*-inositol; cells in panel **b** were grown in minimal medium with 1% gluconate. All scale bars: 5 μm.

To understand the role of the separate Wag31 domains in the cellular distribution of the protein, we expressed different constructs in the *Cglu_P_ino_-wag31* or *Cglu* strains. Heterologous expression of Wag31-mNeon, mNeon-Wag31, or the triple mutant (Ile18-Asp, Lys20-Ser, Arg21-Ser) of full-length Wag31 (Wag31_3mut_-mNeon) in the *Cglu_P_ino_-wag31* strain restored rod shape (Fig. 6a). However, only the C-terminally tagged Wag31-mNeon (i.e., with an unperturbed NtD) phenocopied the slightly asymmetric Wag31 localization to the poles and to the septum that was observed when Wag31-mNeon was expressed in the WT (Fig. 6b). In contrast, either fusing mNeon to the protein N-terminus or introducing a triple mutation in the NtD led to a strong asymmetric polar distribution in *Cglu_P_ino_-wag31* (Fig. 6a), suggesting that these NtD perturbations interfere with the correct cellular distribution of Wag31 in the absence of the endogenous protein. In the WT *Cglu* strain where endogenous Wag31 is present, the heterologous expression of mNeon-Wag31 does not significantly alter polar and septal localization (Fig. 6b). However, the expression of Wag31_CtD_-mNeon (i.e., lacking the NtD) or Wag31_3mut_-mNeon (with a triple mutation at the tip of the NtD) led to a striking unipolar localization and very little signal at the septum (Fig. 6b), again suggesting that a functional NtD is crucial for correct cellular distribution. This unipolar distribution of Wag31 coincides with unipolar PG incorporation (Fig. S4), suggesting that cell wall incorporation at one cell pole is sufficient to maintain rod shape. Finally, this asymmetry also seems to be linked to septum misplacement from a mid-cell localization toward the Wag31-free pole, and the concomitant formation of round mini-cells devoid of DNA (Fig. S5).

## DISCUSSION

In this study, we addressed the domain functionality of Wag31 (DivIVA), a cytoskeletal protein that is largely documented but mechanistically poorly understood. Our structural phylogenetic analysis of DivIVA homologs revealed a clear cutoff in sequence and structure between *Firmicutes* and *Actinobacteria*, which correlates with the different functions that have been associated with DivIVA-like proteins (12) and could explain why DivIVA from the *Actinobacteria S. coelicolor* or *M. tuberculosis* rescued the morphological defect of a *C. glutamicum* strain depleted of Wag31 and restored polar PG synthesis, unlike DivIVA from the *Firmicutes B. subtilis* or *Streptococcus pneumoniae* (10). The molecular break in the form of an intrinsically disordered region, which appeared very early in the actinobacterial tree, separates the NtD septum binding function from the CtD self-interacting function. This separation renders the protein more amenable to conformational regulation, for example, regulating the accessibility of the CtD, which could in turn guide the dynamic properties and the septal/polar distribution of the protein, as described below. This evolutionary insight has led us to probe in more detail the molecular function of each domain and its implications for cell cycle progression.

### Assignment of specific molecular functions to different regions of Wag31

We have shown that the Wag31 NtD is a major determinant for septal recruitment, most likely through protein–protein interactions with GlpR (41), RodA (27), and/or probably other—yet to be identified—partners. Interestingly, the DivIVA NtD in naturally branching *Actinobacteria*, such as *Streptomyces,* seems to have similar recruitment properties, as deletion of the NtD in a fluorescent-labeled *S. coelicolor* DivIVA mutant resulted in much fewer lateral foci of fluorescence compared to the wild-type protein (43), suggesting that the NtD is important for the initial recruitment of DivIVA to new hyphal branching sites. Moreover, in *Firmicutes*, the DivIVA domain of GpsB paralogs—homologous to Wag31 NtD—has been shown to directly interact with PBPs in different species (44, 45) and to form a hub for protein–protein interactions at the septum.

On the other hand, the CtD of Wag31 primarily mediates protein self-assembly at the cell poles and may thus be considered the cytoskeletal domain of the protein. Indeed, Wag31 has a high tendency to self-interact and aggregate through the CtD coiled-coils as fluorescently labeled Wag31 forms very bright foci at the poles, the CtD alone forms single, randomly localized, aggregative spots in depleted *Cglu_P_ino_-wag31* cells, and the full-length protein forms a gel-like material *in vitro*. In agreement with this model, the oligomerization capability of *S. coelicolor* DivIVA, which accumulates at growing hyphal tips, was found to be strongly affected by the deletion of the CtD (43).

### Driving forces for Wag31 cellular distribution and function

**Fig 7:**
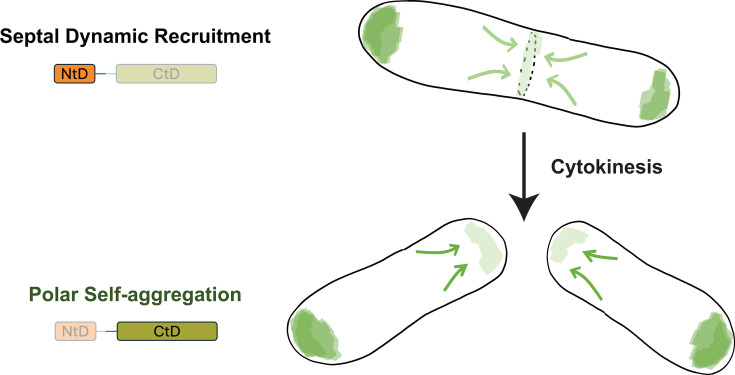
Working model for Wag31 localization in wild-type (WT) and NtD-perturbed cells. In WT *Cglu*, Wag31 is dynamically recruited to the septum *via* NtD-mediated protein–protein interactions, creating a nucleation site for the future pole (top panel). Upon cytokinesis, Wag31 accumulates at the new daughter poles, primarily driven by CtD-mediated self-aggregation (bottom panel). Disruption of the NtD—for example, by introducing mutations in its proximal tip, expressing the CtD alone, or fusing mNeon to the N-terminus—leads to aberrant cellular distribution of Wag31, resulting in strong asymmetry.

Coiled-coil-mediated self-interaction likely drives Wag31 accumulation, acting as a template for further self-assembly. If Wag31 is initially recruited to the septum via the NtD, this septal recruitment could act as a priming mechanism to nucleate Wag31 at the new poles of daughter cells upon cytokinesis. In *Firmicutes*, the main driving force behind DivIVA localization has been attributed to its recognition of negatively curved membranes, although it is unclear how this would work from a mechanistic point of view (13, 14, 46). In *Corynebacteriales*, Wag31 localization appears to be minimally or not influenced by membrane curvature, as shown by the strong asymmetrical localization of Wag31 variants, such as mNeon-Wag31 or Wag31_3mut_-mNeon. These constructs do not equally recognize both cell poles and exhibit reduced septal localization, even if all these regions are negatively curved. Moreover, localization of the Wag31 NtD domain alone to the septum depends primarily on protein–protein interactions, not on membrane binding. Based on our results, we propose a model where the cellular localization of Wag31 in *Corynebacteriales* is instead primarily governed by two key mechanisms: (i) protein–protein interactions that initiate the dynamic recruitment of Wag31 to the septum *via* the NtD, and (ii) auto-accumulation in a concentration-dependent manner that promotes further localization to regions where it is already enriched (Fig. 7). Balance between these mechanisms is essential for proper bipolar and septal localization of Wag31. Disrupting this equilibrium, by interfering with either mechanism, results in aberrant distribution. This model would explain the observed asymmetry when the NtD is altered. Mutations in the NtD, steric hindrance, or domain deletion reduce Wag31 recruitment to the septum, shifting the localization equilibrium toward one cell pole.

The above model emphasizes the importance of understanding Wag31 dynamics to elucidate its function. Given the critical role of the NtD in septal localization, its accessibility likely influences the balanced distribution of Wag31 between the septum and the poles, and *a fortiori* between the old and new poles post-division. The different protein mobility observed between *B. subtilis* DivIVA and *Cglu* Wag31, with the latter being more confined at the poles (40), may stem from differential conformational flexibility and NtD accessibility between *Firmicutes* and *Actinobacteria*, which could alter the accessibility of the coiled-coil domain. In agreement with previous observations (40), our results indicate that there could be distinct Wag31 conformational states within the cell: a dynamic state capable of undergoing subcellular migration to the septum and poles, and a more inert state consisting of higher-order oligomers or aggregates at the cell poles, possibly associated with a different conformational state of the protein (Fig. 7).

### Viability, morphology, and cell envelope biogenesis

Under the conditions used in this study, cells depleted for Wag31 were viable over several generations, in line with a recent report describing a transposon insertion in the *wag31* gene in *Cglu* (47). This was surprising as several past reports indicated that Wag31 is an essential gene in *Cglu* (17), as well as in *Streptomyces coelicolor* (11), *Mycobacterium tuberculosis* (48), and *Mycobacterium smegmatis* (18). These discrepancies may be due to differences in experimental design, cell imaging limitations, and/or a lack of quantitative analysis of the earlier studies. Although Wag31-depleted cells remain viable, capable of dividing and assembling a complete cell envelope, they exhibit significant morphological changes, transitioning from a rod to a coccoid shape. This indicates that Wag31 plays a crucial role in maintaining cell shape and can thus be classified as a morphogenic protein. The rod-to-coccus transition upon Wag31 depletion is likely a result of disrupted polar PG synthesis and regulation. Interestingly, Wag31-depleted coccoid cells can form a septum where they segregate their DNA and synthesize PG and the outer cell envelope components. However, for proper cell division to occur, it seems better to have no Wag31 than to have one with a corrupt NtD, as complementation of Wag31 depletion with constructs containing a dysfunctional NtD led to a strong asymmetry, with unipolar localization, misplaced septa, and anucleate minicells. Wag31 was previously shown to interact with ParB in *Cglu*, which in turn forms the ParB-*oriC* nucleoprotein complex that tethers the chromosome to the cell pole for correct segregation (28). Therefore, a unipolar localization of Wag31 or a dysfunctional NtD could interfere with correct chromosome segregation. It is plausible that the polar Wag31 foci attract and confine ParB-*oriC*—and thus the chromosomes—to one pole only, as the opposite pole has much less Wag31 and would fail to retain the sister *oriC*. As septum placement in the cell was also found to depend on chromosome segregation, through potential nucleoid-associated factors or just steric considerations (49), division site selection would occur where there is no DNA, moving away from the pole where Wag31 accumulates.

Together, our work suggests that actinobacterial Wag31 may be regulated differently from *Firmicutes* DivIVA throughout the cell cycle, in line with its different biological functions. Driving forces other than affinity for negatively curved membranes exist to promote a balanced polar and septal localization, including species-specific interactions with the NtD. Specific regulation of Wag31 oligomerization via protein–protein interactions with other divisome components and/or post-translational modifications might prevent the premature formation of polar-like aggregates at the septum through conformational or electrostatic control. Indeed, it was shown that the serine threonine protein kinase, AfsK, mediates DivIVA phosphorylation, which in turn influences oligomerization, and it was proposed that this may control the assembly or disassembly of multimeric complexes or higher-order structures formed by DivIVA (50). Wag31 is also known to be phosphorylated in *Mycobacteria*, but the effects of phosphorylation on Wag31 assembly at the poles remain unclear (51–53). The dynamic interplay of these different elements during the cell cycle, together with putative interactions with additional regulatory factors yet to be discovered, will ultimately define the molecular properties and cellular roles of actinobacterial Wag31 in cell morphogenesis and polar growth.

## MATERIALS AND METHODS

All bacterial strains, plasmids, and primers used in this study are listed in Table S1 .

### Bacterial strains and growth conditions

*Escherichia coli* DH5α or CopyCutter EPI400 (Lucigen) strains were used for cloning and grown in Luria-Bertani broth or agar plates at 37°C supplemented with 50 μg/mL kanamycin when required. *C*. *glutamicum* ATCC 13032 (*Cglu*) was used as a wild-type (WT) strain. *Cglu* strains were grown in brain heart infusion (BHI) or CGXII minimal medium (54) plus 4% sucrose at 30°C and 100–120 rpm shaking, supplemented with 25 μg/mL kanamycin when required. When specified, CGXII medium was supplemented with 1% gluconate and/or 1% *myo*-inositol.

### Cloning for protein expression in *Cglu*

For ectopic recombinant expression of the different constructs in *Cglu*, we used the pUMS_3 shuttle expression vector (55), in which the gene of interest is placed under the control of *P_gntK_*, a tight promoter that is repressed by sucrose and induced by gluconate (35). Wild-type or mutant versions of *wag31* were assembled in this plasmid by either Gibson assembly, domain deletions by PCR, or site-directed mutagenesis using the primers listed in Table S1.

For cellular localization studies, codon-optimized mNeonGreen was ordered from Genscript and cloned alone (pUMS_17) or fused in frame to the N-terminus (pUMS_21) or C-terminus (pUMS_25) of Wag31 wild-type or mutant constructs, including a ‌flexible linker between the two fused proteins. The plasmids pUMS_175 and pUMS_176 were obtained by deletion of the regions of interest using the plasmid pUMS_25 as a template. The plasmids pUMS_254 and pUMS_255 were generated by site-directed mutagenesis using plasmids pUMS_25 and pUMS_175, respectively, as templates.

### *Cglu_P_ino_-wag31* conditional depletion strain construction

The conditional depletion strain *Cglu_P_ino_-wag31* was generated using a two-step allelic exchange strategy with the suicide plasmid pK19mobsacB (56). In brief, to achieve regulated expression of the *wag31* gene, a transcriptional terminator was inserted upstream of *wag31*, followed by the native promoter of the *ino1* gene (*cg3323*), which is repressible in the presence of *myo*-inositol (33). The transcriptional terminator and *ino1* promoter fragment were PCR-amplified from the plasmid pK19-P3323-lcpA7 (57). Approximately 500 bp regions upstream and downstream of *wag31* were amplified from the genomic DNA of *Cglu*. All DNA fragments were assembled into the pK19mobsacB backbone using Gibson assembly (New England Biolabs).

The plasmid was sequenced and electroporated into *Cglu*. Positive colonies were grown in BHI media supplemented with 25 µg/mL kanamycin overnight at 30°C and 120 rpm shaking. The second round of recombination was selected by growth in minimal medium CGXII plates containing 10% (wt/vol) sucrose. To verify the correct genomic integration of the construct and identify any additional mutations, the wild-type strain was sequenced using both Illumina paired-end and Oxford Nanopore Technologies long-read sequencing. The conditional mutant Pino-wag31 was sequenced using Illumina paired-end. Illumina reads were quality-trimmed and adapter-removed using fastp (v1.0.1). A hybrid *de novo* assembly of the wild-type strain was generated using Unicycler (v0.5.1), combining Illumina and Nanopore reads to resolve repetitive regions. The resulting assembly was annotated using Bakta (v1.12.0). To identify all genomic differences between the wild-type strain and the conditional mutant, Illumina reads from the Pino-wag31 strain were mapped against the annotated parental assembly using breseq (v0.39.0). This analysis confirmed the insertion of the *ino1* promoter region upstream of *wag31*. Four additional SNPs were detected in genes of unrelated function, and the mutations are for amino acids with similar biophysical properties. No other structural rearrangements were identified. All sequencing and analysis data are available as source data.

All the oligonucleotides used to obtain and check this strain are listed in Table S1.

### Western blots

To prepare cell extracts, bacterial cell pellets were resuspended in lysis buffer (50 mM Bis-Tris pH 7.4; 75 mM 6-aminocaproic acid; 1 mM MgSO_4_; benzonase and protease inhibitor) and disrupted at 4°C with 0.1 mm glass beads using a PRECELLYS 24 homogenizer. Total extracts (60 μg) were run on an SDS-PAGE gel, transferred onto a 0.2 μm nitrocellulose membrane, and incubated for 1 h with blocking buffer (5% skimmed milk in TBS-Tween buffer [Tris-HCl pH 8, 10 mM; NaCl 150 mM; Tween-20 0.05% (vol/vol)]) at room temperature (RT). Blocked membranes were incubated for 1 h at RT with the anti-Wag31 antibody (41) diluted in blocking buffer (1:500). After washing in TBS-Tween, membranes were probed with an anti-rabbit horseradish peroxidase-linked secondary antibody (GE Healthcare) for 45 min. For chemiluminescence detection, membranes were washed with TBS-Tween and revealed with HRP substrate (Immobilon Forte, Millipore). Images were acquired using a ChemiDoc MP Imaging System (Biorad).

### Protein expression and purification

6xHis-SUMO-Wag31_NtD_ and 6xHis-SUMO-Wag31_NtD-mut_ were expressed in *E. coli* BL21 (DE3) using an autoinduction protocol (58). Cells were grown in 2YT supplemented with autoinduction medium and 25 µg/mL kanamycin for 6 h at 37°C. After an additional 18 h at 22°C, the cells were collected and frozen at −20°C. The cell pellets were resuspended in 50 mL lysis buffer (500 mM NaCl, 50 mM HEPES, lysozyme, benzonase, EDTA-free protease inhibitor cocktails [ROCHE], pH 8), homogenized, and sonicated. The lysate was centrifuged for 60 min at 8,000 × *g*, filtered through a 0.45 µM surfactant-free cellulose acetate membrane, and loaded onto a 1 mL HisTrap FF Ni-NTA affinity chromatography column (GE Healthcare) using an ÄKTA pure system. The column was washed with 20 mL washing buffer (500 mM NaCl, 50 mM HEPES, 20 mM imidazole, pH 8), and the protein eluted in a linear gradient of elution buffer (500 mM NaCl, 50 mM HEPES, 500 mM imidazole, pH 8). 6xHis-SUMO-Wag31_NtD_- and 6xHis-SUMO-Wag31_NtD-mut_-containing fractions were pooled and dialyzed into protein buffer (150 mM NaCl, 25 mM HEPES, pH 7.8) in the presence of SUMO protease (1:40 protease:protein) at 18°C overnight. The His-tagged SUMO protease and cleaved His-SUMO tags were removed with Ni-NTA agarose resin. After concentrating the cleaved proteins using a Vivaspin 20 3 kDa MWCO centrifugal concentrator (Cytiva) at 12°C, 3,000 × *g*, to a volume of 500 µL, they were loaded onto a Superdex 75 10/300 GL size exclusion column (GE Healthcare) in protein buffer at 4°C. Fractions containing pure Wag31_NtD_ or Wag31_NtD-mut_ were concentrated to 270 µM using an Amicon Ultra 0.5 mL 3 kDa MWCO centrifugal concentrator and stored at −80°C.

Other proteins used for the liposome sedimentation assay (full-length SepF, SepF_Δ14_ lacking the N-terminal membrane-binding segment, and full-length Wag31) were purified as previously described (41, 55).

### Large unilamellar vesicle sedimentation assay

This assay was based on a previously published protocol (30), and large unilamellar vesicles (LUVs) were prepared as described (59–61). In brief, 50 µM protein (Wag31_NtD_, Wag31_NtD-3mut_, SepF, SepF_Δ14_, or Wag31) and 1 mM LUVs (67% PE, 23% PG, 10% CL) were incubated in sedimentation buffer (150 mM KCl, 25 mM HEPES, pH 7.4) for 15 min at 30°C. The samples were centrifuged at 30°C and 100,000 × *g* for 30 min in a TLA55 rotor. The supernatant was collected, and the pellet was resuspended in an equal volume of sedimentation buffer. All samples were CY5 labeled using Amersham QuickStain (Cytiva) before the addition of 4× SDS sample buffer. Samples were denatured for 10 min at 95°C, analyzed by SDS-PAGE, and visualized on a ChemiDoc imaging system (BioRad).

### Phase contrast and fluorescence microscopy

For imaging, cultures of *Cglu* were grown in BHI for around 6 h (except for *Cglu_P_ino_-wag31* strain, which was directly inoculated into CGXII 4% sucrose medium), then pelleted at 5,200 × *g* at room temperature and inoculated into CGXII media supplemented with 4% sucrose and kanamycin (25 μg/mL) for overnight growth. The following day, cultures were diluted to an OD_600_ of 1 in CGXII supplemented with 4% sucrose (with or without 1% gluconate, 1% *myo*-inositol) and grown for 6 h to a required OD_600_ of about 4–6 (early exponential phase). For each sample, 100 μL of culture was pelleted, washed with fresh medium, and diluted to an OD_600_ of 3 for imaging. For PG labeling, HADA stain (Tocris) was added to the culture at a final concentration of 0.5 mM, incubated for 20 min at 30°C and 120 rpm protected from light, and washed two to three times. For Mycomembrane (MM) labeling, 2-Fic5Tre dye (36) was added to the culture at a 33 μM final concentration, incubated for 5 h at 30°C and 120 rpm, and washed four times. For DNA staining, Hoechst stain was added to the culture at a final concentration of 2 ug/mL. For membrane staining, Nile Red (Enzo Life Sciences) was added to the culture (2 μg/mL final concentration) just prior to placing them on 2% agarose pads prepared with the corresponding growth medium. Cells were visualized using a Zeiss Axio Observer Z1 microscope fitted with an Orca Flash 4 V2 sCMOS camera (Hamamatsu) and a Pln-Apo 63X/1.4 oil Ph3 objective. Images were collected using Zen Blue 2.6 (Zeiss), segmented with a custom-trained version of Omnipose (62), and analyzed using the software Fiji (63) and the plugin MicrobeJ (64) to generate violin plots and fluorescent intensity heat maps. Because of the important number of cells analyzed in each sample, Cohen’s *d* value was used to describe effect sizes between different strains independently of sample size:


$$d=\;\frac{mean_{2}-\;mean_{1}}{\sqrt{\frac{\left(n_{1}-1\right)*SD_{1}^{2}+\left(n_{2}-1\right)*SD_{2}^{2}}{n_{1}+n_{2}-2}}}.$$

Values were interpreted according to the intervals of reference suggested by Cohen (65) and expanded by Sawilowsky (66), as follows: very small and small (ns), *d* < 0.5; medium (*), 0.5 < *d* < 0.8; large (**), 0.8 < *d* < 1.2; very large (***), 1.2 < *d* < 2; huge (****), *d* > 2.

### Scanning electron microscopy

*Cglu* and *Cglu_P_ino_-wag31* were grown as described above, harvested in early exponential phase (OD_600_ around 4). The cells were fixed in 2.5% glutaraldehyde overnight at 4°C, and postfixed for 1 h in 1% osmium. Then, samples were dehydrated through a graded ethanol series, followed by critical point drying with CO_2_. Dried specimens were gold/palladium sputter-coated with a gun ionic evaporator, PEC 682. The samples are imaged in a JEOL IT700HR field emission scanning electron microscope.

### Sequence analyses and structure prediction

We assembled a proteomes database containing representative species of all Bacteria obtained from reference 67 (excluding Candidate phyla), five *Firmicutes* model organisms, and 15 representative species covering all actinobacterial diversity (see Data Availability). We used HMM profile searches to identify protein Wag31 in the protein database. First, we used the HMMER package (v3.3.2) (68) tool jackhmmer to look for homologs of *Cglu* Wag31 in all the proteomes using the GenBank (69) sequence BAB99543.1 as query. The hits were aligned with linsi, the accurate option of mafft (v7.475) (70) and default parameters. The alignments were manually curated, removing sequences that did not align globally. The hits obtained by jackhmmer might not include sequences that are very divergent from the single sequence query. For this reason, the alignment was used to create an HMM profile using the HMMER package (v3.3.2) tool hmmbuild. This specific and curated HMM profile of Wag31 was used for a second and final round of searches against the proteomes using the HMMER tool hmmsearch. The new hits were aligned with linsi, the accurate option of mafft (v7.475), and trimmed using trimal (v1.4.rev15) (71) to remove the columns with more than 90% of gaps to facilitate the visualization.

We predicted the structure of the dimeric form of all Wag31 homologs identified in *Actinobacteria* and *Firmicutes* using AlphaFold (72). We used software ChimeraX (73) to map the AlphaFold model confidence on the predicted structures.

### TLC-cell envelope analysis

Lipids were extracted from wet pellets of cells (grown in the stationary phase) with CH_3_OH/CHCl_3_ (2:1 vol/vol) at room temperature for 16 h. The extract was centrifuged for 10 min at 6,000 × *g* to recover the supernatant. After evaporation of the organic phase, the lipids were solubilized once in 1 mL CHCl_3_ and dried again. Finally, the lipid extract was resuspended in CHCl_3_ (generally 50 μL for 20 mL of cells in the stationary phase) and then analyzed by thin-layer chromatography on silica gel (TLC G-60 glass plates, 0.25 mm thick, Macherey-Nagel) developed with CHCl_3_/CH_3_OH/H_2_O (65:25:4, vol/vol/vol). Lipid detection was performed by immersing the TLC plates in 10% H_2_SO_4_ in ethanol, followed by heating to 110°C.

## Data Availability

All phylogenetic data used to produce our results, as well as generated AF models, genome sequences, and assembly, are provided as supporting data under the following link: https://data.mendeley.com/datasets/xtbwh2k7xc/1 (DOI: 10.17632/xtbwh2k7xc.1). All materials of this paper can be provided upon reasonable request. Custom scripts will be made available upon request.

## SUPPLEMENTAL MATERIAL

Supplemental Material mbio.01153-26-s0001.pdf Supplemental figures and table.

ASM does not own the copyrights to Supplemental Material that may be linked to, or accessed through, an article. The authors have granted ASM a non-exclusive, world-wide license to publish the Supplemental Material files. Please contact the corresponding author directly for reuse.
