# Supplementary material for: Functional dissection of Corynebacterium glutamicum Wag31 domains for septal recruitment and polar distribution during the cell cycle
Source: mBio. 2026 Jun 22;17(8):e01153-26. doi: 10.1128/mbio.01153-26 (PMC13463852; doi:10.1128/mbio.01153-26)
Supplement: Supplementary file 1 [file mbio.01153-26-s0001.pdf]

Supplementary Information for:

Functional dissection of *Corynebacterium glutamicum* Wag31 domains for septal recruitment and polar distribution during the cell cycle.

Julienne Petit[1,2], Daniela Megrian[1*], Mariano Martinez[1,2], Adrià Sogues[1], Gabriel Brunk[1], Célia De Sousa-D'Auria[3], Mathilde Ben Assaya[1], Catherine Thouvenot[4], Emilie Lesur[5], Yann Bourdreux[5], Nicolas Bayan[3], Pedro M Alzari[2] & Anne Marie Wehenkel[1#].

1. Institut Pasteur, Université Paris Cité, CNRS UMR 3528, Bacterial Cell Cycle Mechanisms Unit, F-75015 Paris, France
2. Institut Pasteur, Université Paris Cité, CNRS UMR 3528, Structural Microbiology Unit, F-75015 Paris, France
3. Université Paris-Saclay, CEA, CNRS, Institute for Integrative Biology of the Cell (I2BC), 91198, Gif-sur-Yvette, France.
4. Institut Pasteur, Université Paris Cité, Ultrastructural BioImaging Unit, F-75015 Paris, France
5. Université Paris-Saclay, CNRS UMR 8182, Institut de Chimie Moléculaire et des Matériaux d'Orsay, ICMMO, F-91405 Orsay, France.

*Current address: Institut Pasteur de Montevideo, Bioinformatics Unit, 11200 Montevideo, Uruguay.

[#]Correspondance: anne-marie.wehenkel@pasteur.fr

This PDF file includes:

Figures S1 to S5

Table S1

Supplementary References

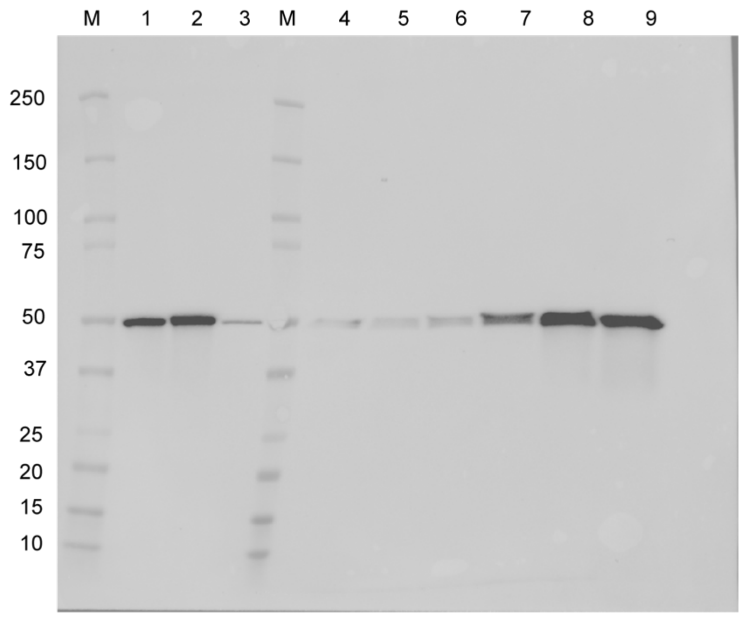

Supplementary Figure S1. *Cglu_P$_{ino}$-wag31* complementation. Western blot of whole cell extracts from *Cglu* (lane 1), *Cglu* + empty plasmid (lane 2) and *Cglu*_P$_{ino}$-*wag31* (lane 3) after overnight growth in minimal medium. Depleted *Cglu*_P$_{ino}$-*wag31* complemented with P$_{gntk}$-*wag31* plasmid in 4% sucrose after 0, 3 and 6 hours (lanes 4-6, respectively) and in the presence of 1% gluconate (overexpression) after 0, 3 and 6 hours (lanes 7-9). Wag31 levels were revealed using anti-Wag31$_{1\text{-}61}$ antibody. Lanes M contain molecular weight markers.

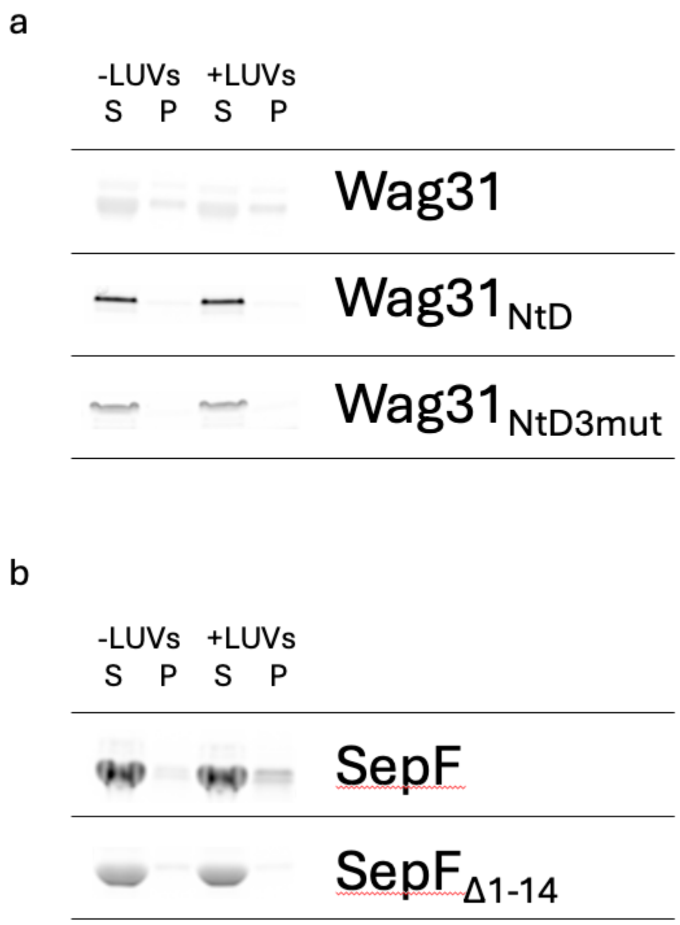

Supplementary Figure S2. **Large Unilamelar Vesicles (LUVs) sedimentation assays.** Sedimentation assay of recombinant Wag31 constructs with 10% cardiolipin-containing LUVs. Crops from SDS-PAGE are superposed vertically for visualisation purposes, separated by black lines. Fractions corresponding to supernatant (S) and pellet (P) are shown in the presence and absence of LUVs. (**a**) Sedimentation results for Wag31, Wag31$_{NtD}$, and Wag31$_{NtD-3mut}$ showing no binding to cardiolipin containing LUVs, in the tested conditions. (**b**) Sedimentation results corresponding to a positive (SepF) and negative (SepF$_{\Delta 1-14}$) control for lipid binding. LUV binding was observed in the positive control (SepF containing a membrane binding sequence segment comprising residues 1-14), but not in the negative control (deletion of the SepF membrane binding domain).

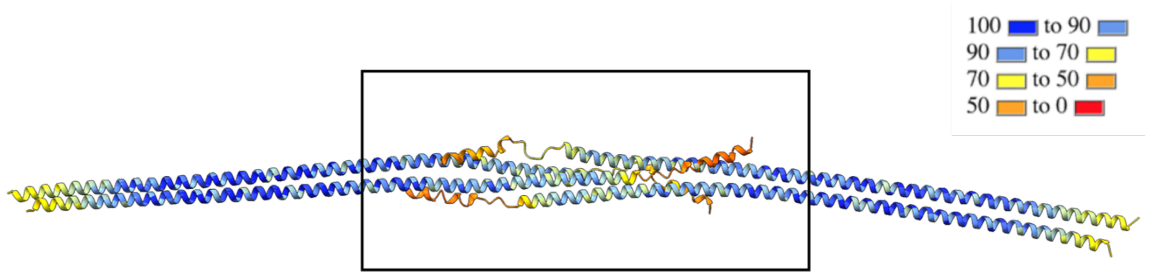

Supplementary Figure S3. AlphaFold model of Wag31$_{CtD}$ tetramerization. Two Wag31 homodimers are predicted to interact with each other through their C-terminal domains (black rectangle). The structural model is color-coded according to model confidence.

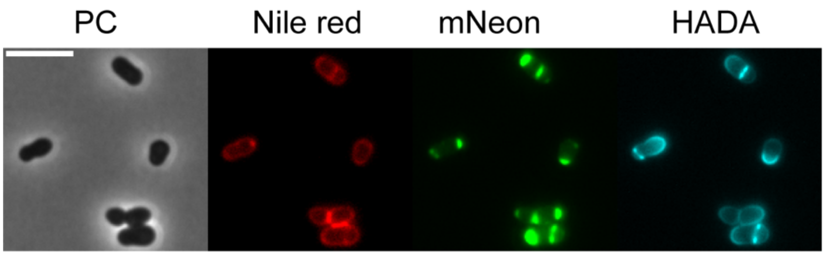

Supplementary Figure S4. Asymmetric peptidoglycan incorporation in cells that display Wag31 asymmetric polar distribution. *Cglu*_P$_{ino}$-*wag31* cells expressing mNeon-Wag31 were stained for membrane (Nile Red) and peptidoglycan (HADA).

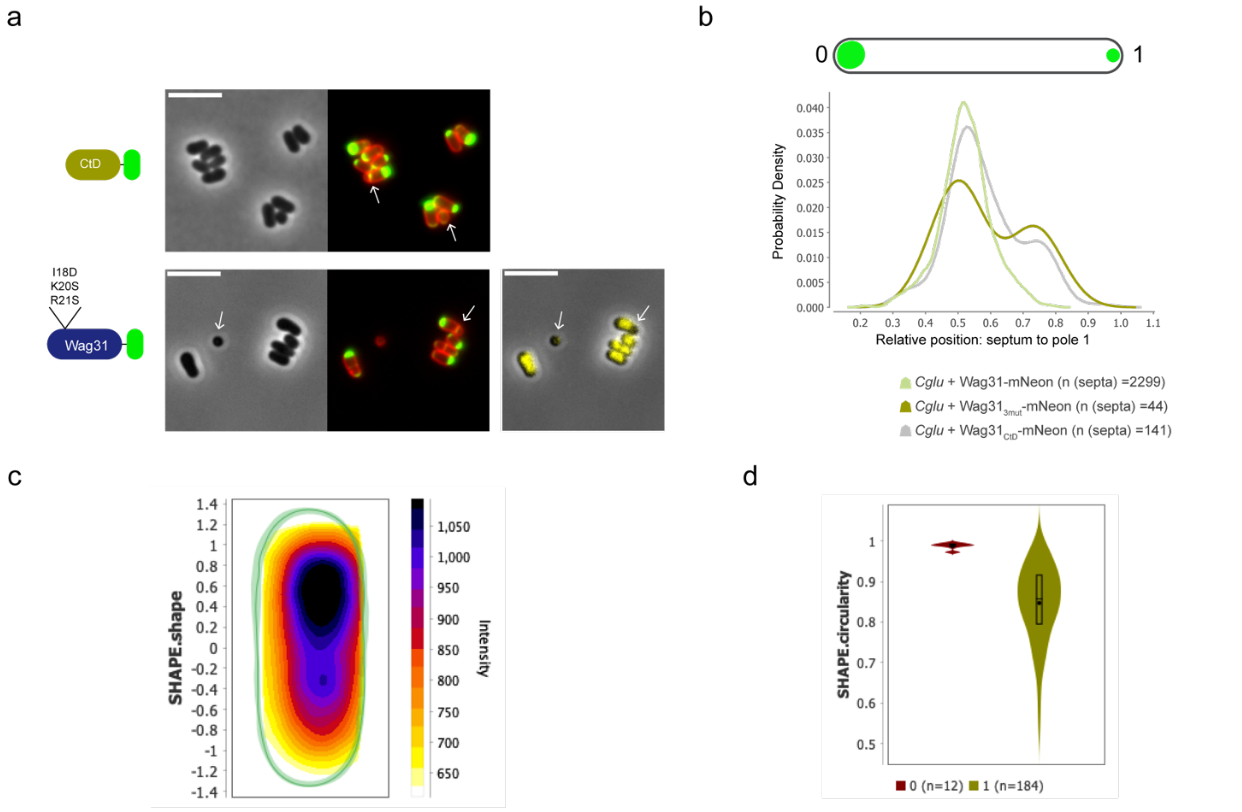

Supplementary Figure 5:. Unipolar localization leads to septum misplacement and creates a population of mini-cells. (**a**) Representative images in phase contrast, Nile Red and mNeon fluorescent signal (red/green overlay), and DNA stain (Hoechst) shown in yellow in *Cglu* expressing the different constructs represented on the left (in minimal medium with 1% gluconate). White arrows indicate mini-cells and misplaced septa. All scale bars 5µm. (**b**) Quantification of septum position in the *Cglu* strain expressing either Wag31$_{CtD}$-mNeon or Wag31$_{3mut}$-mNeon revealed two populations of cells: one with a septum close to mid-cell (relative position close to 0.5, where the pole with Wag31 accumulation is taken as position 0), and one with a relative position around 0.75-0.8. The y axis corresponds to the normalized probability density of septum placement. The lower number *n* of analysed septa for the Wag31$_{CtD}$-mNeon or Wag31$_{3mut}$-mNeon expressing cells is due to a lower number of septa in these cells. (**c**) Heatmap showing asymmetric DNA distribution of *Cglu* + Wag31$_{3mut}$-mNeon cells with one septum (*n*=38). (**d**) Violin plots showing circularity in function of absence (0) or presence (1) of DNA in *Cglu* + Wag31$_{3mut}$-mNeon cells (*n*=196). Note that most cells contain DNA and only few minicells are visible, suggesting either a rare event or a loss of minicells during sample preparation.

Table S1. Bacterial strains, plasmids and primers used in this study.

Strains	Characteristics	Reference
E. coli		
DH5α	F- endA1 Φ80dlacZΔM15 Δ(lacZYA-argF)U169 recA1 relA1 hsdR17(rK–mK+) deoR supE44 thi-1 gyrA96 phoA λ–; strain used for general cloning procedures	(1)
CopyCutter EPI400	F- mcrA Δ(mrr-hsdRMS-mcrBC) Φ80dlacZΔM15 ΔlacX74 recA1 endA1 araD139 Δ(ara, leu)7697 galU galK λ- rpsL (StrR) nupG trfA tonA pcnB4 dhfr; strain used for general cloning procedures	(2)
BL21(DE)	F- ompT hsdSB(rB–mB–) gal dcm (DE3); host for protein production	(3)
C. glutamicum		
ATCC 13032	Biotin-auxotrophic wild type	(4)
Cglu_*P_{ino}-wag31* (sUMS_214)	*myo*-inositol dependent *wag31* silencing strain. ATCC 13032 with insertion of a terminator and P_{ino} promoter to silence *wag31* (*cg2361)* expression. Repressible in the presence of *myo*-inositol	This work

Plasmids for *C. glutamicum wag31* conditional depletion		Reference
pK19mobsacB	KanaR; plasmid for allelic exchange in *C. glutamicum*; (pK18 oriVEc, sacB, lacZα)	(5)
pk19-P3323-lcpA	KanaR; pK19mobsacB derivative. Used as a PCR template to amplify a transcriptional terminator and the promoter of cg3323 (P_{ino})	(6, 7)
pK19-P_{ino}*-wag31*	KanaR; pk19mobsacB derivative containing 500 bp upstream-region of *wag31*, a transcriptional terminator, the P_{ino} promoter and 500bp of the *wag31* coding region	This work

Plasmids for recombinant protein expression in *E. coli*		Reference
pUMS_459	KanaR; pET derivate for $Wag31_{NtD}$ (residues 1-61) recombinant expression containing a N-terminal His-tag followed by a SUMO protease cleavage site	This work
pUMS_460	KanaR; pET derivate for $Wag31_{NtD-3mut}$ (residues 1-61 mutated for I18D_K20S_R21S) recombinant expression containing a N-terminal His-tag followed by a SUMO protease cleavage site	This work

Plasmids for recombinant protein expression in *C. glutamicum*.		Reference
pTGR5	KanaR; *E. coli/C. glutamicum* shuttle vector for regulated gene expression of EGFP under control of tac promoter (Ptac lacI ColE1 oriVEc pGA1 oriVCg)	(5)
pUMS_3	KanaR; pTGR5 derivative in which *Ptac* was exchanged by *PgntK* promoter to control the expression of the EGFP protein	(7)
pUMS_17	KanaR; pUMS_3 derivative for expression of Wag31 under control of *PgntK* promoter	This work
pUMS_25	KanaR; pUMS_3 derivative for expression of Wag31-mNeonGreen (C-terminal tag) under control of *PgntK* promoter	This work
pUMS_21	KanaR; pUMS_3 derivative for expression of mNeonGreen-Wag31 (N-terminal tag) under control of *PgntK* promoter	This work

pUMS_175	KanaR; pUMS_3 derivative for expression of Wag31$_{NtD}$ (residues 1-61) fused to mNeonGreen (C-terminal tag) under control of *PgntK* promoter	This work
pUMS_176	KanaR; pUMS_3 derivative for expression of Wag31$_{CtD}$ (residues 62-365) fused to mNeonGreen (C-terminal tag) under control of *PgntK* promoter	This work
pUMS_254	KanaR; pUMS_3 derivative for expression of Wag31$_{3mut}$, carrying the single mutations I18D, K20S, R21S, fused to mNeonGreen (C-terminal tag) under control of *PgntK* promoter	This work
pUMS_255	KanaR; pUMS_3 derivative for expression of Wag31$_{NtD-3mut}$, carrying the single mutations I18D, K20S, R21S, fused to mNeonGreen (C-terminal tag) under control of *PgntK* promoter	This work

**Oligonucleotide**	**Sequence 5' -->3' and properties[a]**
**Plasmids for *C. glutamicum* strain construction**	
pK19-P$_{ino}$-wag31	
P7_JP	TGTTGTGTGGAATTGTGCCGCTAGGTAATGTGCGC
P8_JP	TTGCGGATTCCCTTCGATTTAACGG
P9_JP	GAAGGGAATCCGCAAATAAAACGAAAGGCTCAGTCGAAAGAC
P10_JP	TGGAGTCAACGGCATCTAAAATTTCTCCTCTTAAAAAGATAACGGCC
P11_JP	ATGCCGTTGACTCCAGCTGATGT
P12_JP	AATTGTTATCCGCTCAGTCCACATTTGCAGCACCTGTAGC
**Plasmids for recombinant protein expression in *E. coli***	
pUMS_459	
P378_MM	GCCACCAATCTGTTCGCGGTGAGCCTC
P379_MM	AGATCCGGCTGCTAACAAAGCCCGAAAGGAAG
P514_MM	GAGGCTCACCGCGAACAGATTGGTGGCATGCCGCTGACCCCGGCGGATGTG
P515_MM	CCTTTCGGGCTTTGTTAGCAGCCGGATCTTTACGCAACCTGCGCCTCCAGTTC
pUMS_460	
P378_MM	GCCACCAATCTGTTCGCGGTGAGCCTC
P379_MM	AGATCCGGCTGCTAACAAAGCCCGAAAGGAAG
P514_MM	GAGGCTCACCGCGAACAGATTGGTGGCATGCCGCTGACCCCGGCGGATGTG
P515_MM	CCTTTCGGGCTTTGTTAGCAGCCGGATCTTTACGCAACCTGCGCCTCCAGTTC

**Plasmids for recombinant protein expression in *C. glutamicum***	
pUMS_17	
P178_MM	ATGGTCTTATCCTTTCTTTGGTGGC

P179_MM	AGCGGCCGCTTAAGGTAC
P216_MM	CCAAAGAAAGGATAAGACCATATGCCGTTGACTCCAGCTGATGT
P218_MM	CGGTACCTTAAGCGGCCGCTTTACTCACCAGATGGCTTGTTG
pUMS_25	
P178_MM	ATGGTCTTATCCTTTCTTTGGTGGC
P179_MM	AGCGGCCGCTTAAGGTAC
P214_MM	CTCGAGGGATCTGGCCAGGGACCGGGCTCAGGCCAAGGAAGCGGCATGGTG TCCAAGGGCGAAGAG
P215_MM	GAATTCGGTACCTTAAGCGGCCGCTTTACTTGTACAGTTCATCCATGCCCATCA CATCGGTGAATG
P216_MM	CCAAAGAAAGGATAAGACCATATGCCGTTGACTCCAGCTGATGT
P217_MM	CCCTGGCCAGATCCCTCGAGCTCACCAGATGGCTTGTTGTTG
pUMS_21	
P178_MM	ATGGTCTTATCCTTTCTTTGGTGGC
P179_MM	AGCGGCCGCTTAAGGTAC
P190_MM	CAAAGAAAGGATAAGACCATATGGTGTCCAAGGGCGAAG
P191_MM	GCCAGATCCCTCGAGCTTGTACAGTTCATCCATGCCC
P212_MM	ACAAGCTCGAGGGATCTGGCCAGGGACCGGGCTCAGGCCAAGGAAGCGGC ATGCCGTTGACTCCAGCTGATGT
P218_MM	CGGTACCTTAAGCGGCCGCTTTACTCACCAGATGGCTTGTTG
pUMS_175	
P13_JP	GGCAACCTGCGCCTCTAGCTCT
P14_JP	GCGCAGGTTGCCCTCGAGGGATCTGGCCAGGGAC
pUMS_176	
P15_JP	CATATGGTCTTATCCTTTCTTTGGTGGCGT
P16_JP	TAAGACCATATGGGTGGTACTTCTTCCGCTGCTAGTT
pUMS_254 and pUMS_255	
P39_JP	CCTGACGGCTCCAGTGGCTACAACGAAG
P40_JP	CACTGGAGCCGTCAGGCGGCTTATTAAAAGC

[a]Overlaps for Gibson assembly or site-directed mutations are highlighted in red

Supplementary References

1. Hanahan D. 1983. Studies on transformation of *Escherichia coli* with plasmids. *J Mol Biol* **166**:557–580.

2. D H. 2004. *Epicentre Forum* **11**:6.

3. Studier FW, Moffatt BA. 1986. Use of bacteriophage T7 RNA polymerase to direct selective high-level expression of cloned genes. *J Mol Biol* **189**:113–130.

4. Shimono SKSUM. 1957. Studies on the amino acid fermentation. *J Gen Appl Microbiol* **3**.

5. Schäfer A, Tauch A, Jäger W, Kalinowski J, Thierbach G, Pühler A. 1994. Small mobilizable multi-purpose cloning vectors derived from the *Escherichia coli* plasmids pK18 and pK19: selection of defined deletions in the chromosome of *Corynebacterium glutamicum*. *Gene* **145**:69–73.

6. Baumgart M, Schubert K, Bramkamp M, Frunzke J. 2016. Impact of LytR-CpsA-Psr Proteins on Cell Wall Biosynthesis in *Corynebacterium glutamicum. J Bacteriol* **198**:3045–3059.

7. Sogues A, Martinez M, Gaday Q, Assaya MB, Graña M, Voegele A, VanNieuwenhze M, England P, Haouz A, Chenal A, Trépout S, Duran R, Wehenkel AM, Alzari PM. 2020. Essential dynamic interdependence of FtsZ and SepF for Z-ring and septum formation in *Corynebacterium glutamicum. Nat Commun* **11**:1641.
